# Hysteroscopy: where did we start, and where are we now? The compelling story of what many considered the “Cinderella” of gynecological endoscopy

**DOI:** 10.1007/s00404-024-07677-x

**Published:** 2024-08-16

**Authors:** Salvatore Giovanni Vitale, Andrea Giannini, Jose Carugno, Bruno van Herendael, Gaetano Riemma, Luis Alonso Pacheco, Amal Drizi, Liliana Mereu, Stefano Bettocchi, Stefano Angioni, Sergio Haimovich

**Affiliations:** 1 Division of Gynecology and Obstetrics, Department of Surgical Sciences, University of Cagliari, Cagliari, Italy; 2 Unit of Gynecology, Department of Surgical and Medical Sciences and Translational Medicine, Sant’Andrea Hospital, Sapienza University of Rome, Rome, Italy; 3 Obstetrics, Gynecology, and Reproductive Science Department, Minimally Invasive Gynecology Division, University of Miami, Miller School of Medicine, Miami, FL USA; 4 Endoscopic Training Center Antwerp (ETCA), Cadix General Hospital, Ziekenhuis Aan de Stroom (ZAS) Antwerp, Antwerp, Belgium; 5 Università degli Studi dell’Insubria, Varese, Italy; 6 Obstetrics and Gynecology Unit, Department of Woman, Child and General and Specialized Surgery, University of Campania “Luigi Vanvitelli”, Largo Madonna Delle Grazie 1, 80138 Naples, Italy; 7 Centro Gutenberg, Unidad de Endoscopia Ginecológica, Hospital Xanit Internacional, Málaga, Spain; 8 Independent Consultant in Obstetrics and Gynecology, Algiers, Algeria; 9 Department of Obstetrics and Gynecology, Policlinico G Rodolico, CHIRMED, University of Catania, Catania, Italy; 10 Department of Obstetrics and Gynecology, University of Foggia, Azienda Ospedaliero-Universitaria “Ospedali Riuniti”, Foggia, Italy; 11 Department of Obstetrics and Gynecology, Laniado University Hospital, Netanya, Israel; 12 Adelson School of Medicine, Ariel University, Ariel, Israel

**Keywords:** Hysteroscopy, Gynecologic surgery, History, Minimally invasive surgery

## Abstract

Hysteroscopy has truly revolutionized the field of diagnostic and operative gynecology. It is presently regarded as the gold standard method for both the diagnosis and treatment of intrauterine diseases and it has fundamentally altered the way gynecologists treat patients with such conditions. These pathologies can now be diagnosed and treated in an outpatient setting, thanks to technological advancements and instrument downsizing. Two hundred years of development and notable innovation are now reflected in the present hysteroscopic practice. This review attempts to trace the boundaries-pushing history of hysteroscopy by highlighting the advancements in technology and the therapeutic and diagnostic benefits offered by this groundbreaking approach.

## Introduction

The introduction of hysteroscopy represents a real revolution in clinical gynecologic practice and has completely changed the way gynecologist approach patients with intrauterine pathology. It is currently considered the standard of care for the diagnosis and treatment of intrauterine pathologies, such as abnormal bleeding, polyps, fibroids, myoma, synechiae, placental remnants, intrauterine device malposition, adenomyosis, congenital malformation and other uterine abnormalities [1, 2].

Hysteroscopy can be diagnostic or operative, offering a panoramic view and the complete visualization of the endometrial cavity [1]. Hysteroscopic technology is evolving and plays an important role in improving women’s health as one of the most commonly performed procedures worldwide.

Thanks to the technical innovations and smaller instruments, the diagnosis and treatment of intrauterine pathologies can now be performed in an office setting [1, 3–6].

The current hysteroscopic practice results from 200 years of development and significant innovation. This review aims to trace the history of hysteroscopy by highlighting the technological progress and the diagnostic and therapeutic capacity provided by this procedure.

## Methods

The present study is a narrative review primarily focused on studies published in the field of history and evolution in hysteroscopy.

In May 2024, an extensive literature search was conducted to identify relevant publications on the most relevant databases (MEDLINE, Embase, PubMed, Google Scholar, and Cochrane). Articles were searched using the following keywords: “hysteroscopy”, “history of hysteroscopy” and “development of hysteroscopy”. No filter on the year of publication was set. The selected articles were rigorously reviewed and evaluated to identify studies that met the aim of this review. The analytic process was completed by reading the full-text version of the articles, categorizing relevant issues, and summarizing the findings. A narrative synthesis of the selected studies was subsequently conducted, integrating further theoretical notions obtained from selected book chapters referenced in the included studies. The evaluation of the narrative review was made according to SANRA guidelines [7].

## Results

### From the dawn of time to the present

#### Hysteroscopic pioneers and first instruments

**Fig. 1:**
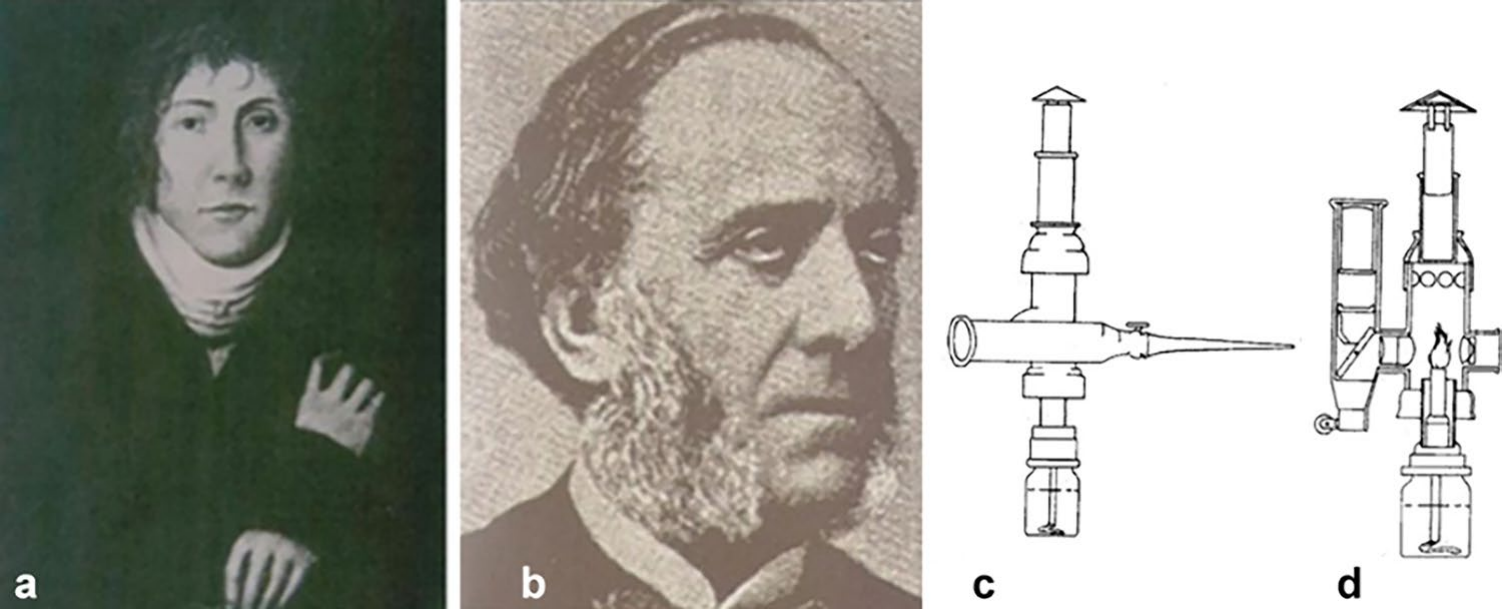
**a** Philipp Bozzini (1773–1809); **b** Antonin Jean Desormeaux (1815–1894); **c** Diagram of Desormeaux’s endoscope **d** Sagittal view showing flame and reflecting lenses

Bozzini (1773–1809) (Fig. 1a) is considered to be the father of endoscopy. In 1804, he developed a light conductor called the *Lichtleiter*, allowing him to visualize the internal cavities of the human body, creating a principle of illumination with a concave mirror [8]. In 1843, the French physician Antonin Jean Desormeaux (1815–1894) developed the first cystoscope (Fig. 1b) by introducing Bozzini’s instrument into the bladder. This device was characterized by a hollowed instrument that allowed direct visualization thanks to a light produced by an alcohol and turpentine lamp that passed through one-half of the tube before being reflected by a concave mirror inserted into a viewing tube [8]. His endoscope was a system of mirrors and lenses with an open flame as the light source, and by filling the bladder with fluid, it was possible to observe the cavity through a glass window (Fig. 1c-d).

It was not until 1869 when the application of urological principles was translated to the uterine cavity: Pantaleoni (1810–1885) was the first to perform a hysteroscopic procedure. He diagnosed a small endometrial polyp in a 60-year-old woman suffering from postmenopausal bleeding and succeeded in treating it with repeated cycles of silver nitrate, using Desormeaux’s instrument [9].

**Fig. 2:**
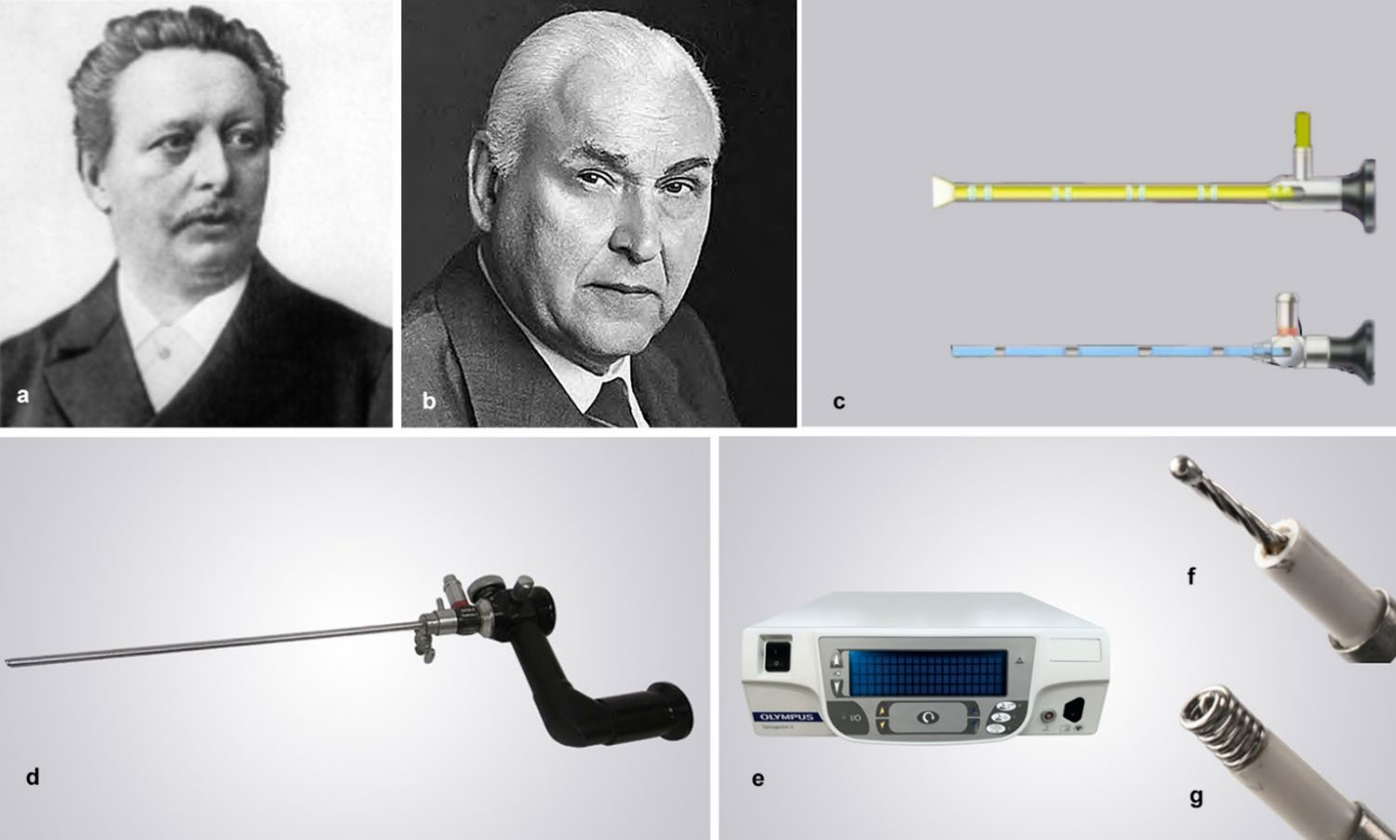
**a** Maximilian Carl­Friedrich Nitze, (1848–1906) **b** Karl Storz (1911–1996; **c** Small spherical lenses (yellow) vs cylindrical lenses (blue); **d** Type 1 Hamou Microcolpohysteroscope; **e** Versapoint II™ Bipolar Electrosurgery System (Bipolar Electrosurgery System, OLYMPUS Europa SE & Co.KG); **f** Versapoint II™ Twizzle bipolar electrode; **g** Versapoint II™ Spring bipolar electrode (color figure online)

Although closely related to cystoscopy, innovation in hysteroscopy progressed at a slower rate. This is likely due to difference in properties between the uterine cavity and the bladder, with the uterus being less complaint and more difficult to distend, more fragile and more prone to bleeding. The cystoscope soon became a widely used practice after its introduction by Nietze in 1879 (Fig. 2a) [8]. He was the first to combine light and lens, but this innovation in the urological field was not accepted by the gynecologic community at the time.

In addition to the difficulty of exploring the uterine cavity, the first instruments had a diameter of about two cm, causing intense pain to patients when introduced into the uterus.

In 1907, David developed the first contact hysteroscope, thinking that a view in direct contact with the surface of the endometrium would be an important advance for the observation of the postpartum or post-abortion uterus [10].

Because of the need to distend the uterus to observe its cavity, several ingenious modifications to the first hysteroscope followed. Heineberg, in 1914, created the first continuous flow hysteroscope, adding a parallel channel for the instillation of heated saline solution, allowing a constant flow of the distention media [10].

The use of carbon dioxide to distend the uterus was introduced by Rubin during the early 1900s, but its use was not adopted. Most physicians preferred to work with low-viscosity fluids [8].

After numerous attempts, Norment, in North Carolina, developed his own continuous flow hysteroscope [8]. However, it was Silander in 1960 who popularized this technique, allowing an accurate view of the endometrium but only for diagnostic procedures. Meanwhile, Quinones-Guerrero and collaborators added a 5% water solution of dextrose while modernizing Norment’s method with special tourniquets and pumps to increase intrauterine pressures and to improve the continuous flow [8].

#### Revolutionary years: 1959–1990

The latter half of the twentieth century proved to be crucial and revolutionary in the development of endoscopy. The changes which took place shaped the technique to what we are accustomed with in our current day-to-day practice [11–13].

While the group of Aguero in Venezuela focused on adapting the use of flexible gastroscopes to visualize the uterine cavity in pregnancy, in 1959, the German entrepreneur and inventor Karl Storz (1918–1994) in collaboration with Hopkins (1911–1996), focused their attention on lenses, generating a real revolution in modern endoscopy (Fig. 2b).

By changing the lens from a spherical one to a longer cylindrical one, they managed to improve the brightness and sharpness of the image while minimizing its deformation. Furthermore, this change allowed for a modification in the shape and length of the device and reduction in the caliber of the instrument (Fig. 2c) [9].

In Japan, Mohri, using a flexible hysteroscope, pioneered the use of cameras to dynamically evaluate his observations, paving the way for the extraordinary film recordings made by Norment Muller and Keller from the mid-1950s to the mid-1970s. He was primarily concerned with the observation of embryos in early pregnancy and documented their early movements [8].

Moreover, in 1968, Menken was the first to propose a complete endoscopic evaluation of the female genital tract, from the vagina to the uterus [9]. He also was the first to propose using high-density fluid, emphasizing its smaller amount needed and the reduced risk of peritoneal spread. It was not until 1970, however, that Edstrom and Fernström used dextran solution with a molecular weight of 70,000 for hysteroscopy. They appreciated its viscosity, transparency, and ability to keep the cavity distended without causing bleeding. Their original report established the routine use of high-viscosity dextran as distention media for hysteroscopy [9, 14].

The role of Lindemann (1920–2012) in 1971, in Germany, was very important since he proposed the use of carbon dioxide (CO_2_) for intrauterine distension. He proved that a flow rate of 80–100 mL/min and an insufflation pressure of no more than 200 mmHg were safe and useful for the procedure [15–19].

In 1974, Lindemann, in collaboration with Wiest, designed the Hysteron-insufflator, making the control of the pressure of the insufflated gas possible. This device includes adaptable flow and insufflation settings. They also solved the problem of having the gas escape during the procedures, creating a special cervical adapter consisting of a suction hood [20–23].

In the Japanese article titled *Diagnostic and Therapeutic Hysteroscopy* published in 1978, it was Sugimoto who first proposed the use of saline solution as a distension medium. However, the problem of obtaining a continuous flow irrigation system that would allow a clear view while avoiding excessive vascular intravasation of the fluid remained. It was established that the resistance of the uterine vasculature should not be overcome by the pressure of the infused intrauterine fluid. To avoid fluid overload, new equipment, such as electronic pumps, designed to calibrate the rate of fluid infusion and the pressure exerted in the intrauterine cavity, were introduced. The main safety factors were: paying close attention to details during the procedure, controlling the intrauterine pressure of the fluids, minimizing endometrial/myometrial vascular damage, and limiting the duration of the procedure [8].

The real revolution in the hysteroscopic field occurred at the end of the 1970s and the early 1980s, by Hamou; he designed the first Hamou Microcolpohysteroscope (Fig. 2d). This revolutionary instrument had a diameter of 5 mm and allowed four different powers of image magnification up to 150 times, which was great for cellular exploration, allowing both contact and panoramic hysteroscopy. In 1987, he published a book on hysteroscopy and microcolpohysteroscopy (Hysteroscopie et Microcolpohysteroscopie–Atlas et Traité) which became a guide in the field of hysteroscopy, describing CO_2_ distension and the 30° fore-oblique view of the optic, which enabled the visualization of the entire uterine cavity only by rotating the scope without lateral movements. Hamou, besides a gynecologist, being also a polytechnician and engineer, also rebuilt the insufflator systems for CO_2_ hysteroscopy as well as the pumps for liquid distention: the Hamou Endomat systems.

More complex operating procedures were performed starting in the 1980s, aided by the introduction of the video camera and the use of unipolar electrosurgical systems. These ensured less technical difficulties in guiding the instruments: one hand (passive hand) held the video camera up to the eye, while the other (the operating hand) was able to make free movements and perform the surgical procedure more easily [24, 25].

Hysteroscopists thus began to perform even the most demanding operative procedures, such as lysis of uterine septa, polypectomies, and surgical removal of small intracavitary myomas. Surgeons moved from operations with mechanical instruments to the use of unipolar energy, with the introduction of hysteroscopic resection as already adopted by urologists. In 1975, Iglesias and his helpers published a report on their resectoscope, which has emerged as the first prototype of ‘modern’ resectoscope. The Iglesias resectoscope permitted simultaneous suction and continuous irrigation, which results in better visualization. In 1976, Neuwirth and Amin described the first case of excision of submucous myomas using a urologic monopolar resectoscope. Since that time, hysteroscopic myomectomy has been developed and has become a safe and efficient procedure [26].

In 1987, Hallez designed the first resectoscope specifically for gynecologic use; it included a 6.5-mm-diameter sheath, a 3-mm scope (0° angle view), and a continuous flow system that used a mannitol–sorbitol or glycine solution with ethanol—to detect fluid overload by breathalyzer—as the distention medium of the uterine cavity, approved by the Food and Drug Administration (FDA) in 1989. Hysteroscopic procedures thus began to enter the routine clinical practice of gynecologic surgery. Thanks to technological innovations, the procedures quickly improved year by year. These include continuous flow systems for operative and diagnostic hysteroscopes, vaporizing bipolar electrodes (Fig. 2e-g), and miniaturization of instruments for professional use [9, 27–30]. Among all the practitioners, former American Association of Gynecologic Laparoscopists (AAGL) medical director Löffer and Valle were among the most enthusiast hysteroscopic pioneers, showing how modern hysteroscopy should prevail over conventional dilation & curettage (D&C) for accuracy and feasibility [30]

#### The turning point: Bettocchi

The brilliant Italian Bettocchi had a great impact on the clinical use of hysteroscopy, with the innovative concept of office hysteroscopy [31, 32].

**Fig. 3:**
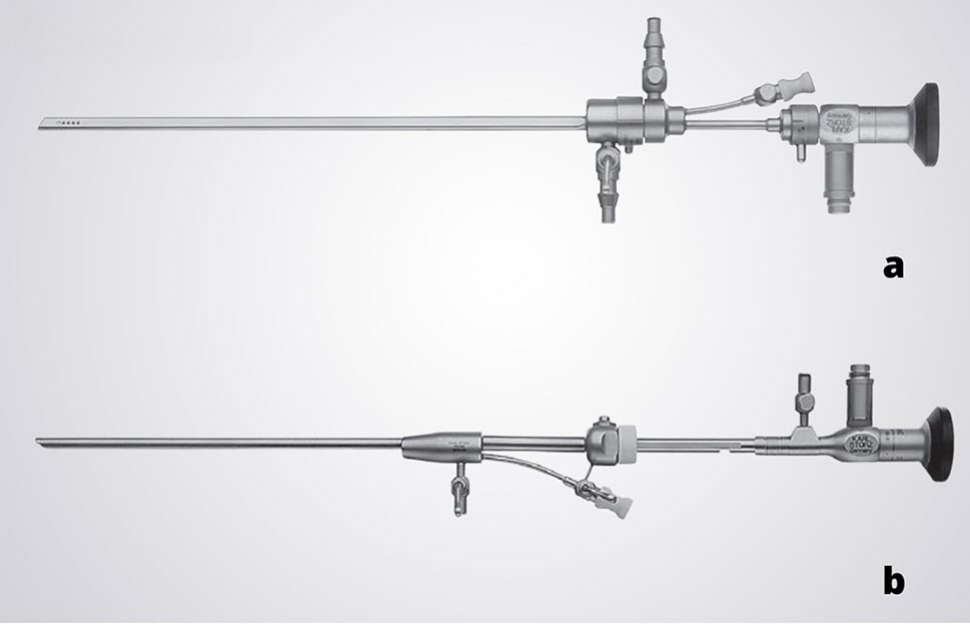
**a** Continuous flow hysteroscope acc. Bettocchi diameter 5 mm (Karl Storz Endoskope, Tuttlingen, Germany); **b** TROPHY® scope compact hysteroscope acc. Campo (Karl Storz Endoskope, Tuttlingen, Germany)

He developed small-diameter hysteroscopes with continuous flow of distention media and operative channels, through which miniaturized instruments can be inserted. The oval sheet with a cross section and a diameter of only 5 mm was modified from the double-sheathed continuous flow 6.5-mm chorionscope of Antonio Perino to make it more ergonomic and easier to insert through the cervical canal. In addition, the hysteroscope had an operating channel that could accommodate up to 5 Fr instruments (Fig. 3a). Also, he played an important role in the growing acceptance of normal saline solution as the primary distention medium for uterine cavity, which later replaced the CO_2_ [33].

Bettocchi developed a new painless method for introducing the hysteroscope into the uterine cavity: the vaginoscopy or no-touch technique. This technique allows the introduction of the hysteroscope inside the vagina and inspection of the vaginal canal, the external cervical os, the cervical canal, the internal cervical os and the uterine cavity, avoiding the need to introduce a speculum and/or use a tenaculum and making it possible to investigate an organ previously approachable only with manual palpation [34–39].

In these ways, Bettocchi contributed significantly to the development and spread of ambulatory hysteroscopy and to the diagnosis and the treatment of pathologies in the office setting (the so-called see & treat approach) [1, 2, 39, 40]. The underlying concept is that the diagnostic and operative intervention can be performed in a single procedure [41].

In 1995, Mazzon from Rome described the cold loop myomectomy, which allowed the mechanical enucleation of intramural submucosal myomas taking into account the myoma pseudocapsule, changing the approach to this pathology [9].

Bipolar electrosurgery was first used in hysteroscopy in 1997 with the creation of the Versapoint.™ Bipolar Electrosurgical System, first introduced by the company Gyrus subsequently bought by Gynecare (Johnson & Johnson, New Brunswick, NJ, USA) and subsequently acquired by Olympus (Olympus America CORP. USA) after commercialization of the version II (Fig. 2e-g) [37]. It allows the use of normal saline as the distension media instead of non-conductive solutions (glycine, sorbitol–mannitol, etc.). It also has the added benefit of reducing tissue energy loss during resection [2, 9].

#### New hysteroscopic operating techniques

The classic technique to remove uterine intracavitary pathologies, such as endometrial polyps and submucous myomas, is resectoscopic hysteroscopy (HR), which requires the use of monopolar or bipolar high-frequency energy allowing to excise intrauterine pathology.

Mark Hans Emanuel created a new instrument: the Intra Uterine Tissue Retrieval System (IUTRS) (prototype; Smith & Nephew Endoscopy, Andover, MA), with the aim of creating a procedure that is easier to learn and safer [42].

In 2007, the first pilot approaches and subsequent studies on the application of diode laser in hysteroscopic surgery were made by Sergio Haimovich. The main advantage of such technique was the improved hemostatic power, reduced thermal dispersion and minimal damage to surrounding healthy tissues [4].

IUTRS, as the Truclear (Truclear, Medtronic INC, USA), consists of a set of two metal, hollow, rigid, disposable tubes that fit into each other; the two tubes rotate over each other. The rotation is controlled by a foot pedal driven mechanically by an electrically powered control unit; both tubes have a window-opening at the end with cutting edges. Using a vacuum pump connected to the inner tube, the resected tissue is then aspirated through the device into a collecting pouch for histopathologic investigation.

The device (4 mm in diameter) is introduced into the uterine cavity with a custom-designed 9-mm rigid, continuous flow with a 0° hysteroscope.

In 2009, the MyoSure® (Hologic, Marlborough, USA) was released into the market, with different measures, a smaller 2.5-mm inner blade that rotates and reciprocates within a 3-mm outer tube, at a higher speed [43]. Then the Truclear 5.0 hysteroscopy system (Medtronic INC, Minneapolis, USA), incorporating a 2.9 mm rotary-style morcellator through a 5 mm, 0° hysteroscope was proposed [44].

In 2010, Giampietro Gubbini from Bologna, Italy developed the first continuous flow-through mini-hysteroscope (Tontarra Medizintechnik GmbH, Wurmlingen, Germany). This 21-French resectoscope can be used with monopolar or bipolar energy. Later, a 17- and a 15-French resectoscope were developed.

Also, in 2011, an evidence-based guideline covering best practice in outpatient hysteroscopy (OPH) was published by the Royal College of Obstetricians and Gynecologists (RCOG) [45].

Rudi Campo from Belgium developed the Trophyscope (Campo TROPHYSCOPE®) (Karl Storz SE & Co KG Tuttlingen Germany) (Fig. 3b) between 2013 and 2014, a diagnostic instrument with an outer diameter of only 2.9 mm that can be turned into a 4.4 mm operative hysteroscope.

Another useful IUTRS was created by Giuseppe Bigatti, who developed the 24 French and later the 19 French Integrated Bigatti Shaver IBS $\circledR$ (Karl Storz SE & Co KG Tuttlingen, Germany). These instruments also allow the passage of sturdy instruments 7 French and electrical loops.

All these innovations led to an optimal use of the hysteroscopic technique both in diagnosing and in treating the patient in an ambulatory setting.

#### Hysteroscopic miniaturized instruments

The introduction of miniaturized resectoscopes and hysteroscopic IUTRS, eliminating the need to dilate the cervix mechanically or pharmacologically, has revolutionized the world of hysteroscopy [47, 48]. Modern hysteroscopes are currently available with an outer diameter smaller than 6 mm, which can be introduced into the uterine cavity without dilating the cervix.

IUTRS have become a fundamental part of the instruments used for ambulatory operative hysteroscopy [49]. However, what remains fundamental for hysteroscopy is the never-aging possibility of performing eye-targeted endometrial biopsies.

Endometrial biopsy is considered the gold standard for the diagnosis and treatment of uterine factor infertility, for the study of abnormal uterine bleeding and the diagnosis of endometrial hyperplasia and cancer, among other intrauterine conditions [50, 51].

The macroscopic identification of focal abnormalities suggestive of endometrial hyperplasia/cancer within the uterine cavity to be visualized and targeted for biopsy under direct visualization is a strong advantages for OPH [1].

There are various types of hysteroscopic forceps that differ in their tip end and allow to perform endometrial biopsies. A good endometrial biopsy depends on the type of instrumentation used, endometrial characteristics and technique of the surgeon.

The standard technique is defined as “the punch biopsy”, in which the jaws of the biopsy forceps grasp the endometrium. Its use is now limited because the collected tissue volume of tissue is minimal and often insufficient for an adequate histopathological diagnosis. In 2002, Bettocchi introduced a new technique, known as “grasp biopsy” [46–50]. It is characterized by toothed grasping forceps (alligator forceps), and it is one of the most used in current clinical practice. This kind of forceps consents to add further endometrial tissue that surrounds the forceps and protrudes from it. In the case of multiple biopsies, its utilization is limited. In this case, the surgeon needs to insert, remove, and reinsert the hysteroscope multiple times, which would cause discomfort to the patient [50, 51].

**Fig. 4:**
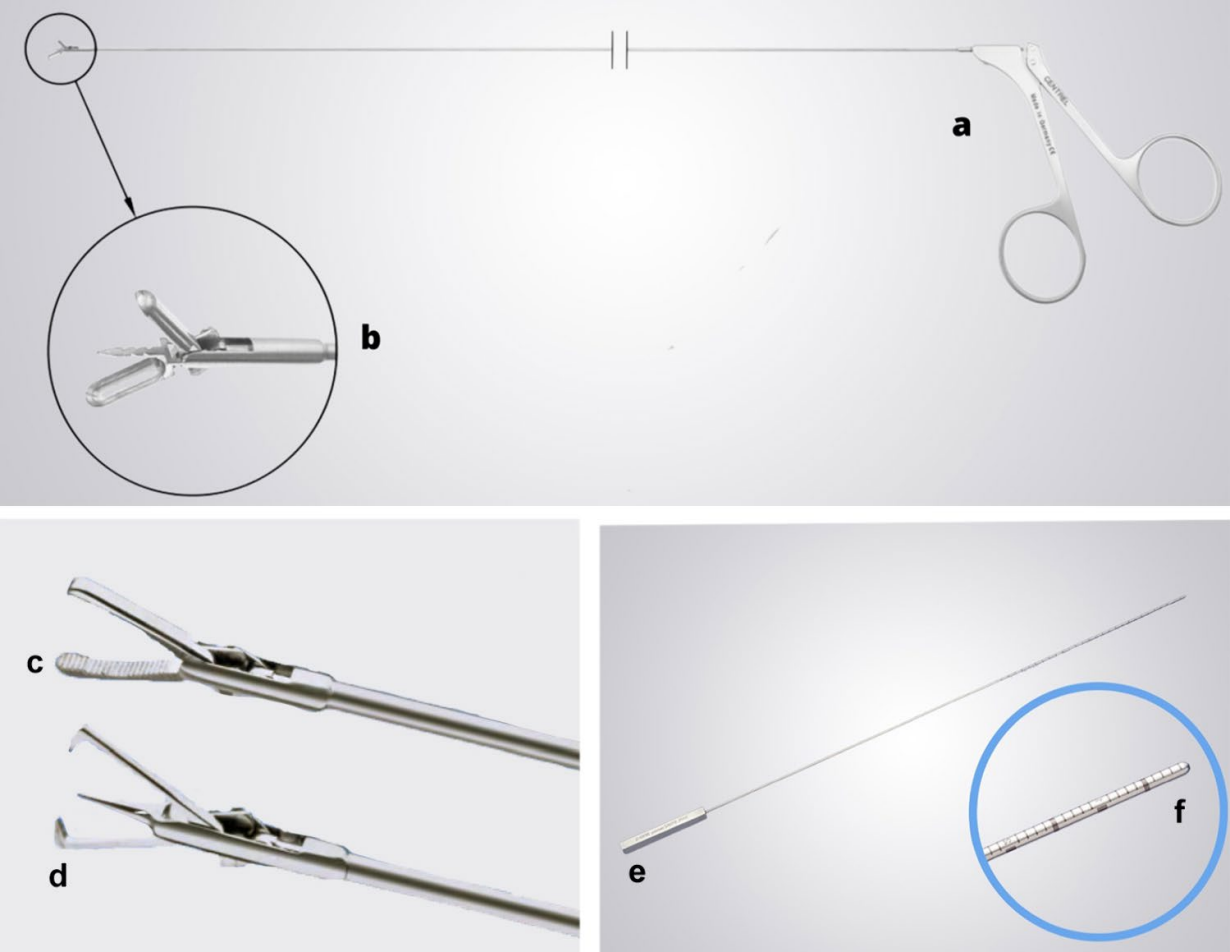
**a** Snake biopsy forceps acc. Vitale (Biopsy Snake Grasper sec. Vitale. Centrel S.r.l., Ponte San Nicolò, Italy); **b** Focus on the tip of the instrument; **c** 5 Fr mechanical instruments designed to facilitate the removal of fragments of tissue from the uterine cavity: Di Spiezio Sardo grasping forceps; **d** Hesseling / Di Spiezio Sardo tenaculum grasping forceps with spike**; e** Uterine palpator acc. Bettocchi/Di Spezio Sardo (Karl Storz Endoskope, Tuttlingen, Germany); **f** Focus on the end of the instrument

In 2020, Vitale introduced an innovative instrument for endometrial biopsy, the biopsy snake grasper sec. Vitale (Centrel Srl, Ponte San Nicolò, Italy), which can be used to grasp and cut at the same time [52]*.* It is a robust, easy-to-use tool compatible with all modern hysteroscopes equipped with a 1.67 mm (5 French) working channel (Fig. 4a, b). To have introduced this significant innovation, this young Italian researcher was awarded the “Netter Prize” by the European Society of Gynecology in 2023.

This method allows biopsy to be performed even on thin endothelium, such as atrophic endometrium. It is made feasible by the sharp edge’s ability to cut through even flat surfaces and the tip's ability to prevent loss of the specimen. The problem of discomfort for some patients who need repeated biopsies could still limit the method [50].

Two other five Fr instruments are available in the office hysteroscopic setting—Di Spiezio Sardo grasping forceps and Hesseling/Di Spiezio Sardo tenaculum grasping forceps (Karl Storz SE & Co KG Tuttlingen, Geramny) with spike designed to facilitate the removal of fragments of tissue from the uterine cavity (Fig. 4c, d).

For hysteroscopic metroplasty, the graduated intrauterine palpator is used, specifically designed to measure the length of the uterine cavity, of the cervical canal and the resected septum (in millimeters/centimeters) (Fig. 4e, f).

**Fig. 5:**
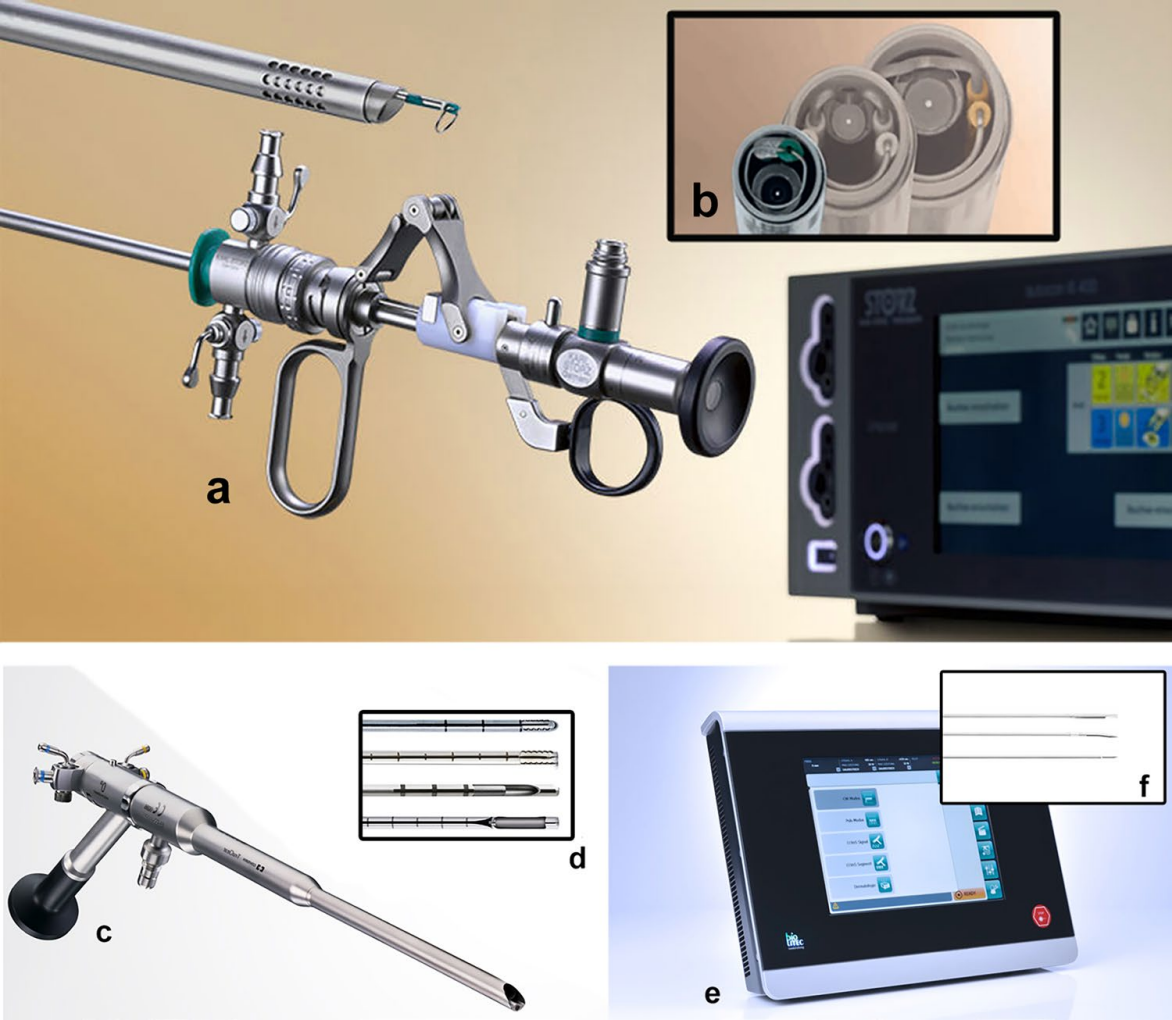
**a **16 Fr Mini-Resectoscope (Karl Storz SE & Co.KG, Tuttlingen, Germany). **b** Focus on the caliber of the device. **c** TruClear™ hysteroscopic tissue removal system (Medtronic INC, Minneapolis, USA). **d** Available blades. **e** Leonardo® Dual-wavelength Leonardo Laser System (D.w.L.S.; Leonardo, Biolitec, Germany. **f** Focus on available blades

A very commonly used instrument is the Twizzle bipolar electrode. It allows to perform the “Chips Biopsy”, as well as the 16-Fr miniresectoscope (Fig. 5a, b).

When atrophic or hypertrophic endometrium is present in perimenopausal and postmenopausal women, deeper endometrial biopsies can be performed with this approach, which is especially helpful in situations where using mechanical forceps would not be adequate to obtain enough tissue for diagnosis. The main disadvantage of this technique is, notably, the need of bipolar electrodes, which are more expensive than mechanical forceps and have the risk of thermally damaging the surrounding endometrium and the tissue collected for biopsy [50–53].

The introduction of an IUTRS (Fig. 5c, d) allows for the “Visual D&C”. The cut/aspiration mode allows a systematic sample of a large portion of the uterine cavity, making it an effective alternative to conventional operative resectoscopy [54]. The biggest limit of this method is the inability to actively coagulate bleeding vessels and the high cost.

In conclusion, it can be stated that the choice of tool and technique used for hysteroscopic endometrial biopsy, including miniaturized mechanical grasp forceps, miniresectoscopes or diode lasers (Fig. 5e, f) depends on the type of pathology, operator's experience, and comfort with the technique.

#### Present: a new universal language for hysteroscopy

Currently, office hysteroscopy is the gold standard for the diagnosis and treatment of intrauterine diseases while hysteroscopic septum resection is the standard of care for this pathology. The specific clinical case determines which therapeutic technique is the best choice, it is important that we all use the same terminology when referring to hysteroscopic procedures [9].

#### International training centers and International societies sharing information, recommendations, and guidelines.

In 2009, Arnaud Wattiez—at that time, the president of the ESGE—invited Bruno J van Herendael to produce a document “Training in Endoscopy”. This 47-page document approved by the Board of the ESGE became the fundamental basis of the Gynaecological Endoscopic Surgical Education and Assessment (GESEA) training program. GESEA, being the first real teaching system as far as endoscopy, is concerned in the world. In the following years, due to the invaluable use ultrasound has in diagnostics and operative guidance, it became evident that it should be integrated into the curriculum. In 2019, the Endoscopic training Center Antwerp (ETCA) started with highlighting the link between ultrasound and hysteroscopy, to emphasize on the necessity for proper preoperative diagnosis before embarking in a more invasive technique. This initiative (Bart De Vree, Hysteroscopy & Ultrasound—Building Bridges) set the step to an integrated ambulatory see and treat approach.

In 2021, a working group of experts on hysteroscopy, the AAGL, the European Society for Gynaecological endoscopy (ESGE) and the Global community of hysteroscopy (GCH) responded to the need for the implementation of a common terminology to describe hysteroscopic procedures, to be used worldwide both in clinical practice and in the research setting [55].

The aim was the creation and adoption of a standard nomenclature that could be adopted by surgeons, patients, and researchers. With that in mind, a consensus statement of recommended terminology for use when describing hysteroscopic procedures such as pain management, the setting where procedures are conducted, the model of care relating to the length of stay and need for admission, the type of procedure, instruments, and the approach to hysteroscopy was created [56]. This further emphasized the notion to consider hysteroscopy a sub-specialty within its right, requiring surgeons with a particular skill set and specific training. Since then, the GCH organizes his “Global Congress on Hysteroscopy” every two years, and several initiatives have been deployed by different societies, the latest being HARTUS in 2024 (Hysteroscopy, Assisted Reproductive Technology and Ultrasound), the SONOMIGS™ of the International Society for Gynecologic Endoscopy (ISGE), bringing together high-end ultrasound and hysteroscopy was launched as a worldwide initiative in 2024. The aim of the latter is to train surgeons in first a basic and thereafter in advanced courses both theoretical and practical ending with exams. These concepts [57–65] and trainings [66–70] allow for a continuous interaction and debate between specialists.

### What’s next?

The main of innovation in the field is to reduce the diameter of the instrument, to improve patient experience and safety, while improving on image quality without increasing the cost [62].

#### Digital hysteroscopy clinic

The Digital Hysteroscopy Clinic (DHC) is a new concept in precision medicine, which is based on the fusion of outpatient operative hysteroscopy and the high-level technology typical of modern operating rooms [1, 63]. It is ideal for a one-stop diagnostic procedure and surgical approach, putting the patient at the center of diagnostic and therapeutic [1, 63].

The pioneer of the Digital Hysteroscopy Clinic is Rudi Campo from Belgium, who integrated 3D sonography into the endoscopic tower in 2018, representing the birth of DHC [2].

Maintaining a technologically advanced and well-organized structure is crucial, both for outpatient and digital hysteroscopy clinics. To reduce the woman's discomfort, the team should provide the space as comfortable as possible [1]. This is critical to minimize the risk of complications and to maximize the cost-effective utilization of medical resources [9, 64].

DHC allows the treatment of 90% of intrauterine pathologies. It must always be highlighted that an ASA grade > 2 or the presence of large lesions that require lengthy surgery and large instruments cannot be treated with DHC and must be treated in the operating room [1, 9].

#### 3D Hysteroscopy

3D hysteroscopy is still a fresh concept. The addition of depth perception to an image is the principle in its conversion from 2 to 3D. The primary benefits of 3D endoscopy are increased safety, accuracy, and precision during operation, as well as the preservation of tactile feedback sensation [71]. Furthermore, because of its enhanced hand–eye coordination, this technique offers a low learning curve. Because 3D hysteroscopy uses the same devices as 2D hysteroscopy, it is still a relatively affordable alternative. Adding 3D image visualization could completely change the way we think about hysteroscopy, helping additional insights also in proximal tubal patency, uterine malformations or retained product conception evaluation and removal, and offer up new avenues for gynecologic endoscopy research [72–78].

#### Hysteroscopy and artificial intelligence (AI)

One of the main limitations of hysteroscopic-assessed pathologies is the subjectivity of the diagnosis before histopathological confirmation. To increase its diagnostic accuracy, AI, machine learning, and deep learning model could improve sensitivity while maintain high specificity [79]. Deep learning models can provide higher performances in classifying endometrial images compared to operators. However, larger databases and further comparative trials, especially in case of premalignant and malignant lesions, are needed to validate the available findings [79].

#### Robotic hysteroscopy

Considering over 200 years of progress and considering where all the surgical fields are heading to, it won’t be surprising that robotic hysteroscopy seems a future target. Harvey et al. [80] developed a prototype for a 23Fr rigid system based on concentric tube robots enabling two-handed surgery in small spaces in a simulation laboratory. This was a feasibility study of the endoscopic robot for hysteroscopic applications, including removal of a simulated endometrial polyp, Asherman’s syndrome and other intrauterine diseases. Potential advantages of this approach could be to the improved exposure, finer dissection capability, and use of two-handed surgical maneuvers. However, smaller caliber and flexible robotic instruments could be even easier to apply in clinical practice, demanding additional trials before the application in human settings [80].

#### Training for future generation

The recommendation of standards of training in gynecological endoscopy and related specialties such as hysteroscopy are important objectives of the international societies, such as AAGL, ESGE, GCH and ISGE. All these programs lead to bringing theoretical and practical skills to surgeons involved in hysteroscopy both diagnostic and operative.

The aim of the National and International societies is to form the new generation of hysteroscopists and nurses to be able to tackle the complaints of the patients in an office setting with minimal discomfort for the patient and optimum environment for the operator.

## Conclusion

Over the last 200 years, the art of hysteroscopy has developed and evolved through the work of numerous pioneers whose work and interventions have allowed advancement in both the technological and technical aspect of the field.

Hysteroscopy is a sub-specialty of gynecological endoscopy that changed the approach to uterine pathology, thanks to good visualization, satisfactory diagnosis, and treatment of pathologies. The need for highly specialized gynecological operators is therefore mandatory, and it is increasingly easier with the development of modern, ergonomic, and practical tools to constantly improve the patient experience.

## Data Availability

No new data were created or analyzed in this study.
